# Training and Competition Load Monitoring and Analysis of Women's Amateur Basketball by Playing Position: Approach Study

**DOI:** 10.3389/fpsyg.2018.02689

**Published:** 2019-01-09

**Authors:** María Reina Román, Javier García-Rubio, Sebastián Feu, Sergio José Ibáñez

**Affiliations:** ^1^Grupo de Optimización del Entrenamiento y Rendimiento Deportivo (GOERD), Universidad de Extremadura, Cáceres, Spain; ^2^Facultad de Educación, Universidad Autónoma de Chile, Santiago, Chile

**Keywords:** performance analysis, women, basketball, internal load, external load

## Abstract

Currently, the number of women involved in sport is increasing. Although, research on their characteristics and performance is scarce. A great amount of research on men's basketball is available, but it is unknown if it can be applied to women's basketball. The objective of this research was to characterize the internal and external load performed by female basketball players during training and sports competition according to playing positions through inertial devices. The participants in the following study were 10 amateur basketball players who competed at regional level (21.7 ± 3.65 years; 59.5 ± 12.27 kg, and 168.5 ± 3.56). Data were collected in games of the final phase (*n* = 8) and from 5 vs. 5 training tasks (*n* = 47). All the analyses were run according to playing positions. Each player was equipped with a Garmin^TM^ Heart Rate Band and Wimu^TM^ inertial device that monitored physical activity and movement in real time. The results obtained showed that the load experienced during competition was significantly higher (*p* < 0.001) than during training (Heart Rate, Player Load, Steps, Jumps, and Impacts). There were also differences according to playing positions, mainly between the backcourt and frontcourt players (*p* < 0.001). The players must work in higher areas of heart rate during training, mainly in Z4 and Z5, increasing their HRmáx y HRavg. The training doesn't equal the load supported and the distance performed in competition, so it is necessary to pay more attention during training. This information allows us to develop adequate training protocols adjusted to the specific individual requirements of the sports competition.

## Introduction

The load experienced by athletes in competition is one of the most important research topics in sports science. Knowing the physical and physiological demands of sports competition is the key to determining optimum training processes (Torres-Ronda et al., 2016). The physical demands of team sports are difficult to quantify due to the unpredictable and intermittent nature of the sport (Caetano et al., 2015), in basketball, intense actions are combined with periods of rest, and these do not follow temporal patterns. There are studies seek to understand what happens during competition in order to replicate it in training; however there are few studies that compare what happens in training with what happens in actual competition (Reilly et al., 2009).

Previous research has already reported the different methods used to quantify and measure load in training and matches, mainly employing internal training load analysis (iTL) and external training load (eTL) measurement by Time Motion Analysis (Matthew and Delextrat, 2009; Scanlan et al., 2012). With external training loads representing the physical work performed during the training session or internal training loads that are the biochemical (physical and physiological) and associated biomechanical stress responses (Impellizzeri et al., 2005). Besides, associations between internal and external measures of load and intensity are important for understanding the competition and training process (Vanrenterghem et al., 2017).

Different means exist to facilitate the description and analysis of the load that athletes endure in training and competition. The monitoring of external load measurements derived from triaxial accelerometers is currently considered a viable tool in indoor team sports (Chambers et al., 2015). In turn, the use of micro-technological sensors such as accelerometers has also allowed the analysis of body movements representing an unspecific assessment of the quantity and magnitude of high-intensity activities without measurable movements on the court surface (i.e., jumps, screens, rebounds, etc.,) which can't be registered by GPS (Puente et al., 2017). From a practical point of view, the use of technology in official competitions allows a more accurate control of the athlete's load during a match, providing an important reference for training prescription besides allowing trainers and scientists to identify the level of fatigue by detecting the changes in individual players' movement mechanics due to fatigue (Barrett et al., 2016; Barreira et al., 2017).

Basketball is a sport that comprises complex technical-tactical abilities that have a direct influence on the physiological requirements imposed on the player during competition (Drinkwater et al., 2008; Ziv and Lidor, 2009). There are many studies which consider that it is hybrid in nature, where most of the energy mobilized has an aerobic origin (McInnes et al., 1995; Abdelkrim et al., 2007; Narazaki et al., 2009). However, as occurs in other collective sports, the explosive actions, such as direction changes, jumps, or maximum intensity movements, as well as some specific game actions, such as shots, getting unmarked, or dribbling, are anaerobic dependent and are decisive for the final performance of the athletes (McInnes et al., 1995; Ostojic et al., 2006; Chaouachi et al., 2009; Narazaki et al., 2009).

Sallet et al. (2005) carried out a study with French basketball players, and concluded that the anaerobic capacity could be considered to be one of the most important performance factors in this sport, regardless of the fact that, quantitatively, aerobic work was more important for the energy supply. The study of the load experienced by the athlete helps to understand the efforts that lead to different energy requirements. The iTL and eTL of basketball players makes it possible to understand the training process, its effects on the athlete and the validity of specific internal measures (Weaving et al., 2014; McLaren et al., 2017). In men's basketball, intensity differences have been found in the play actions regarding the player's position (Abdelkrim et al., 2007). In team sports, it is essential to identify the most appropriate exercise to promote the best performance adaptations. For this, the differentiation of specific game positions is necessary (Stiffler et al., 2015; Vitale et al., 2016, 2018). In women's basketball, differences in the distance covered have been found according to teams' styles of play, but not as a function of match periods (Conte et al., 2015). Besides, it has been confirmed that physiological demands are different according to competitive level and playing position (Rodriguez-Alonso et al., 2003).

With regard to the external load, the distance covered by the female basketball players is similar (5–6 km per match) but there are differences among categories relating to intensity. The players in higher categories spend more time, in an intermittent fashion, in the more intense work zones (Scanlan et al., 2012). During a match the female players carry out between 576 and 652 movement patterns, which implies a change every 3 s (Matthew and Delextrat, 2009; Conte et al., 2015; Delextrat et al., 2015). Regarding the most explosive actions in the game, the players carry out between 1 and 2 jumps per minute and about 45 sprints per match (Matthew and Delextrat, 2009; Conte et al., 2015; Delextrat et al., 2015). In relation to the internal load in women's basketball, the authors have found a mean HR of 162–173 bpm, a HRmax of 188–195 bpm and a %HRmax of 82.4–92.5% (Rodriguez-Alonso et al., 2003; Matthew and Delextrat, 2009; Scanlan et al., 2012; Abad et al., 2016; Reina et al., 2017). With respect to training, there are no ecological studies that assess it, without the intervention of the researcher, although it is considered essential to carry out this type of studies.

When analyzing sports competition together with training, it has been found that the type of training situations has been evaluated in order to compare them with demands generated by competition and in an attempt to try to match them. However, it has only been found that the demands of training equal or exceed those in matches or conditioning exercises (Petersen et al., 2011). Reina et al. (2017) showed that kinematic demands, as well as the more intense cardiac values, depend on the type of playing situation, the loads more similar to the competition are given in the 5 vs. 5 training tasks; however, higher values were always recorded in competition.

In addition, the literature establishes the importance of psychological factors such as motivation, stress or fatigue in sports performance. Coaches use technology to track players throughout the season, real-time monitoring provides data to minimize factors such as fatigue and the risk of injury (Fox et al., 2017). Marcora et al. (2009) state that mental fatigue is a psychobiological state caused by prolonged periods of demanding cognitive activity and is characterized by subjective feelings of fatigue and lack of energy that could decrease performance.

Research that describes women's competition is relatively scarce (Matthew and Delextrat, 2009; Narazaki et al., 2009; Scanlan et al., 2012). It is not clear how to address the individual needs of female players in order to optimize performance while following the principles of sports training, such as individuality and specificity (Boyle et al., 1994; Bompa and Haff, 2009). There are no studies about the work base performed during training, and no studies have been done comparing the load variables recorded by inertial systems, between training and official competition in women's basketball. Thus, the main goal of this research was to analyze the existing differences in the iTL and eTL between a 5 vs. 5 match in an official competition and 5 vs. 5 in female basketball training, as a function of the specific position of each player. Secondly, to know which physical and physiological capacities are the most influential for performance during competition. It was hypothesized that the training load was different from the competition load, being superior in competition. The same would be true of the specific playing positions, each one will have specific characteristics.

## Materials and Methods

### Experimental Approach to the Problem

This investigation follows an associative strategy (Ato et al., 2013) where an attributive variable is utilized and differences between groups are examined. It is a longitudinal observational study performed with amateur athletes participating in official Spanish Regional competitions.

**Figure 1:**
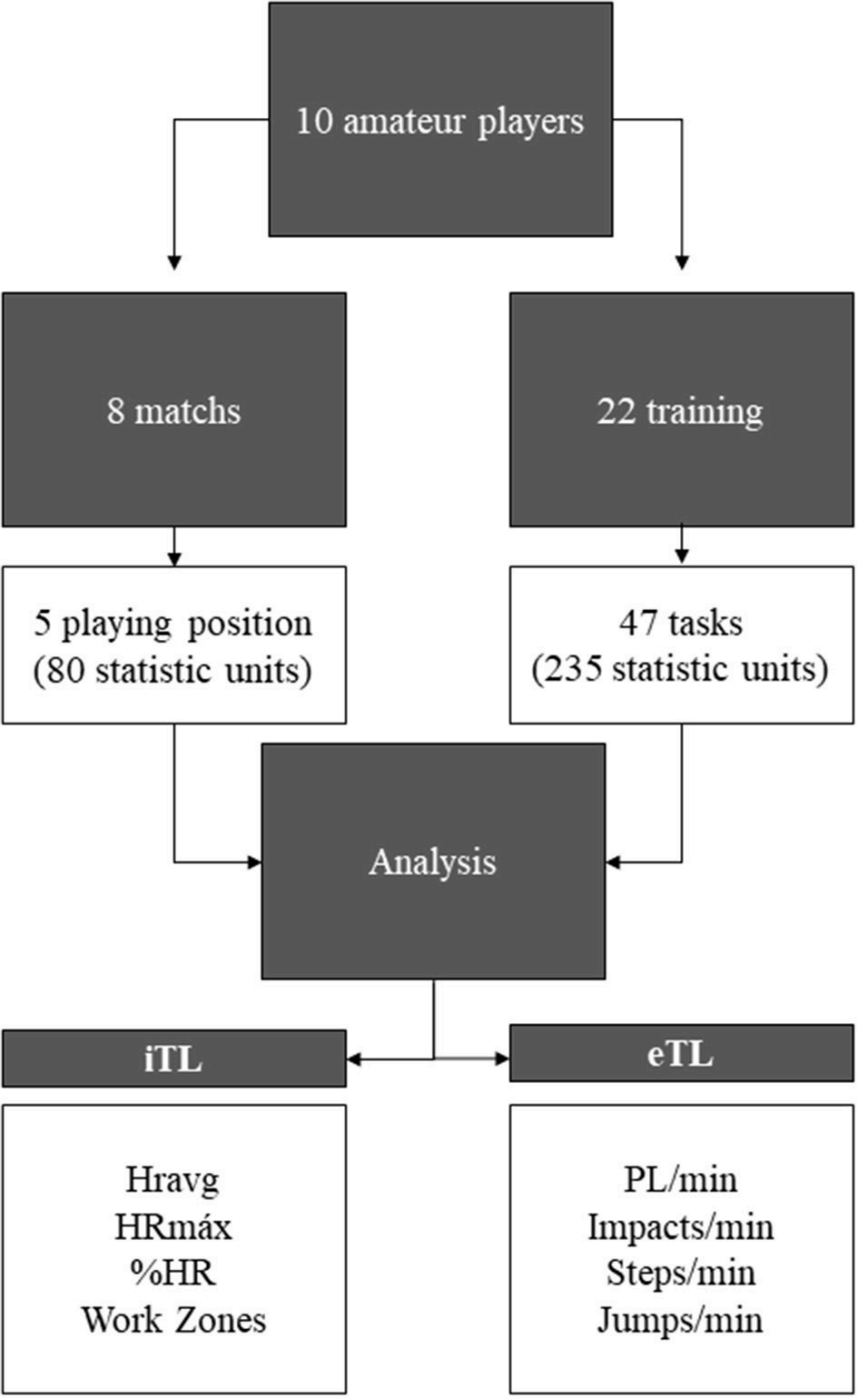
Methods flow chart.

The data was collected after the qualifying phase of the competition. Only games played during the final phase (last 2 months of the season) were analyzed. In this phase, teams of similar characteristics compete among themselves (the six best teams in competition). All players took part in the pre-seasons conditioning program to ensure a good standard of fitness at the start to the championship. Competition habits were established by this time, therefore avoiding the possibility of untrained athletes (Matthew and Delextrat, 2009). In addition, data was collected from training carried out during this period. All the analyses were run according to playing positions. Overall, data sets were collected from 22 training sessions and eight official competitive matches (Figure 1). Only the 5 vs. 5 tasks were used for the analysis, therefore, 47 tasks per position were used (235 statistic units). The purpose was to analyze and compare the full game situation in training and competition. Knowledge of the load that female basketball players reach in training compared with competition, would allow coaches to design sessions with the specific demands of competition for each playing position.

### Subjects

The population to which this study was oriented were senior female Spanish basketball players (>18 years). The team is amateur because they don't receive remuneration. It is a senior team, in which each player has already defined its predominant role in the game. It is true that the general game dynamics may cause them to perform other specific functions during training and competition. The participants in the following study were 10 basketball players (2 Point Guards, 2 Shooting Guards, 2 Small Forwards, 2 Power Forwards, and 2 Centers) who compete at regional level (21.7 ± 3.65 years; 59.5 ± 12.27 kg and 168.5 ± 3.56). All the players belonged to the same club and team, and completed an 8-week pre-season conditioning program consisting of a combined training plan of agility, plyometric, anaerobic, and endurance components, ensuring adequate fitness for the beginning of the competitive season. All players and trainers were informed about the research protocol, requisites, benefits, and risks, and their written consent was obtained before the start of the study. The ethics committee of the University of Extremadura approved the study (n° 67/2017).

### Instruments

To collect the iTL and eTL variables, each player was equipped with a Garmin^TM^ Heart Rate Band and a Wimu^TM^ inertial device (Muyor et al., 2017) that monitors physical activity and movement. The SPro^TM^ software automatically analyzes all the data gathered by the inertial device and sends it to the computer screen in real time. The Wimu^TM^ inertial device was turned on and placed in a specific customized vest pocket located on the posterior side of the upper torso fitted tightly to the body, as is typically used in games. Both the inertial device and the software come from the same organization (RealTrack Systems, Almería, Spain).

### Variables

The independent variable was Game situation (GS), defined as the specific demands and the number of players involved in the training situation (Ibáñez et al., 2016). The 5 vs. 5 training situations with the characteristics of real competition, Full Game (FG), and 5 vs. 5 in official league games (C) were used for the analysis. In addition, four dependent variables were established, iTL; eTL; playing position and time.

#### Internal Training Load

These variables were collected from a heart rate monitor. Heart Rate (HR) was measured in beats per minute. The values were expressed as: Average Heart Rate (HRavg), Maximum Heart Rate (HRmax), %Maximum Heart Rate (%HRmax) and Work zones (Makivić et al., 2013). The work zones were the time that players spent in each zone during the task. Work zones were established as the percentage of the maximum heart rate that each game situation implied. The work zones were Z1 (50–60%), Z2 (60–70%), Z3 (70–80%), Z4 (80–90%), Z5 (90–95%), and Z6 (>95%). These work zones are calculated automatically and individually by the SPRO^TM^ software. For this, the HRmáx presented by each player in each session is taken into account.

#### External Training Load

These variables were recorded with the accelerometers using WIMU^TM^ inertial devices. The specific software SPRO^TM^ processes data raw from the accelerometer automatically. This step avoids possible errors by researchers. The following neuromuscular variables were selected:

- Impacts: Measuring the g force to which the body is subjected in the different play actions being the vector sum of the g forces that a player endures in the three planes (x, y, z). The value of an impact is established when the G force of the movement is higher than 5 Gs (Puente et al., 2017). The manufacturer's software (SPRO^TM^) uses these reference measurements.
- Steps: Movement that implies advancing with a flight time of <400 ms.
- Jumps: Movement that consists in elevating oneself from the court with an impulse that implies more than 400 ms of flight time before landing again, in the same or another place. The manufacturer's software (SPRO^TM^) reference measurements have been used (Pino-Ortega et al., 2018).
- Player Load: Is a vector magnitude derived from the triaxial accelerometry data that quantifies movement with high resolution. It is the vector sum of the accelerations of the device on its three axes (vertical, antero-posterior and lateral), and is calculated from the following equation where (Z) is acceleration on the antero-posterior axis, (X) is acceleration on the medium-lateral axis; (Y) is acceleration on the vertical axis, (t) is time and (n) is number. Accelerations and decelerations are used to build a cumulative measurement of the acceleration change rate. We use a cumulative measurement (PL) and an intensity measurement (PL.min-1), which can, therefore, indicate the stress rate that the player's body is subjected to during a determined time period. The Player Load as a load unit has a moderate-high grade of reliability and validity (Barreira et al., 2017).

(1) $$\begin{array}{l} PlayerLoad_{t=n}\overset{t=n}{\underset{t=0}{\sum }}\sqrt{{\left(Z_{t=i+1}-\;Z_{t=i}\right)}^{2}+{\left(X_{t=i+1}-\;X_{t=i}\right)}^{2}+{\left(Y_{t=i+1}-\;Y_{t=i}\right)}^{2}} \end{array}$$

**Equation 1**. Accumulated PlayerLoad^TM^ used in the quantification of loads in sport. Where: Z, acceleration of the anterior-posterior axis; X, acceleration of the medial-lateral axis; and, vertical axis acceleration; t, time; n, number.

#### Playing Position

Specific position of the player in the team (Point Guard, Shooting Guard, Small Forward, Power Forward, Center).

#### Time

Time in minutes in each 5 vs. 5 training and competition. Rest periods between quarters and time out were excluded from the study.

The data obtained from the selected kinematic variables for this study come from the internal sensors of an inertial device (accelerometers, pedometers, radiofrequencies) and do not rely on data from global positioning systems (GNSS), since for this study the position of the players on the court was not analyzed. For the statistical analysis, all the kinematic data were normalized to the practice time (repetitions per minute).

### Procedures

Once the sample and the competitive phase were selected, data was collected by monitoring each of the players in every training session and matches played during that period. All players took part in the pre-season conditioning program to ensure a good standard of fitness at the start of the competition, avoiding the possibility of having untrained athletes (Matthew and Delextrat, 2009). Players always trained and played in their specific position. The data collection in training and competition was not performed in the same manner, differing in the following way:

#### Training Analysis

Each training week included 3 sessions of an hour and a half each, totaling four and a half weekly training hours, plus the corresponding match. All training sessions in the final competition phase began with 15 standardized minutes based on dynamic stretching exercises, reactivation and racing. The players were allowed to drink water during recovery periods. All training sessions were designed, directed and supervised by the coaching staff. The training sessions were based principally on contesting shot exercises, small sided games, tasks with number superiority or inferiority and 5 vs. 5 tasks. In this study, the demands generated by the FG in training were analyzed to subsequently compare it with the real game. 5 vs. 5 tasks in training took place at the end of the session, with an average total duration of 18.76 min. The tactical instructions were the same as those performed in matches and fluctuated depending on the opponent (Abdelkrim et al., 2010). It has been stated that time-motion variables do not vary according to different defensive tactics (Sampaio et al., 2016). The tasks of 5 vs. 5 respected the rules of the competition and the scoreboard was used to control time, fouls, free-throws.

#### Match Analysis

A real time analysis was made for the four quarters in every competitive game, excluding the rest intervals between quarters (Torres-Ronda et al., 2016). Training and competitive 5 vs. 5 quarters lasted 10 min. Quarters lasted a total of 16 to 19 min. Only the players on the court were analyzed. Analyses were performed during the whole time the quarter lasted. All variables were normalized to minutes.

### Statistical Analyses

First of all, the normal distribution of the data was analyzed with the Kolmogorov-Smirnov test (Field, 2009), to select the subsequent statistical analysis. Next, the kinematic variables were normalized to action per min, due to the difference shown in the time duration of determined tasks and compared to competition. A descriptive analysis of the data was performed with means and standard deviation of all the collected variables in the study both in training and competition. Next, a one-factor ANOVA, with the effect size according to Cohen's *d*, was used to identify the differences between groups and the effect magnitude of training or competition. Effects sizes were calculated by Cohen's *d* from the F-test where effect sizes of 0.20 are small, 0.50 are medium and 0.80 are considered large (Thalheimer and Cook, 2002). Statistical analyses were performed using SPSS v.21 software (Inc., Chicago, IL, USA). Statistical significance was set at *p* < 0.05.

## Results

**Figure 2:**
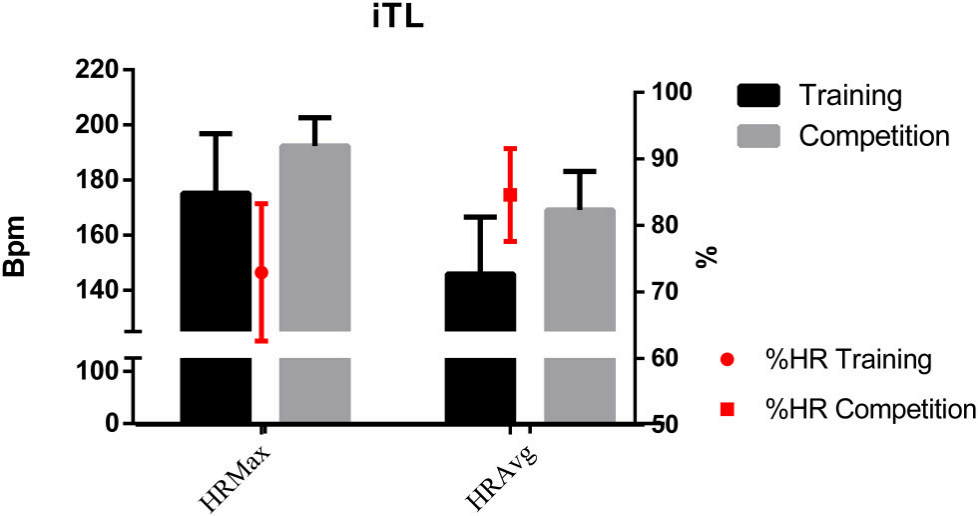
Internal load by game situation.

**Figure 3:**
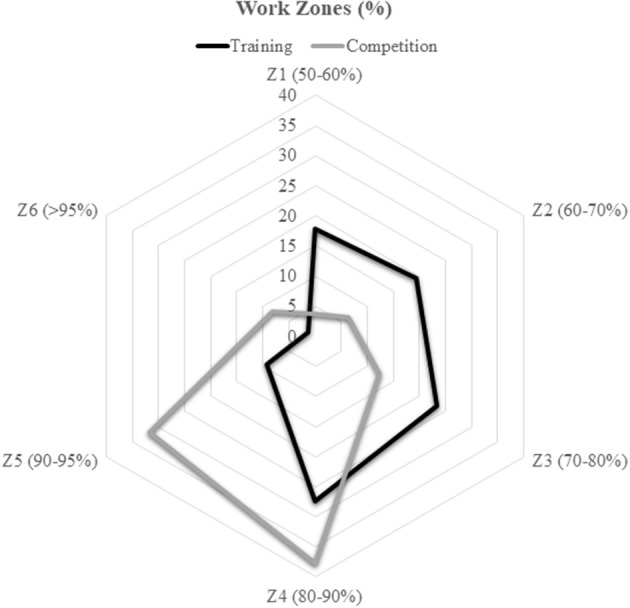
Work zones by game situation.

Table 1 presents the descriptive and inferential results of all variables according to Game Situation (5 vs. 5 in training and competition). The main descriptive results show the higher demand of official matches, as reflected by the results of the iTL variables (maximum, average, heart rate, or the time percentage spent in each zone) (Figures 2, 3).

**Table 1:** Descriptive and inferential results as a function of game situation.

		**5 vs. 5 in Training**	**5 vs. 5 in Competition**	***F***	***d***
iTL	HRMax	175.18	192.33	33.23^***^	1.01
	HRAvg	145.91	169.18	65.16^***^	1.32
	%HRMax	72.95	84.59	65.16^***^	1.32
	Z1 (50–60%)	17.78	3.66	23.83^***^	0.84
	Z2 (60–70%)	19.32	6.30	44.47^***^	1.13
	Z3 (70–80%)	23.28	12.35	26.42^***^	0.92
	Z4 (80–90%)	27.38	37.74	15.89^***^	0.62
	Z5 (90–95%)	9.19	31.84	130.92^***^	1.64
	Z6 (>95%)	1.27	8.09	69.53^***^	0.87
eTL	PL/min	0.94	2.82	814.84^***^	2.37
	Impacts/min	1.69	1.65	0.02	0.02
	Steps/Min	39.15	53.96	63.37^***^	1.15
	Jumps/Min	1.43	1.76	5.12^**^	0.32

* p < 0.05;

** p < 0.01;

*** *p < 0.000*.

**Figure 4:**
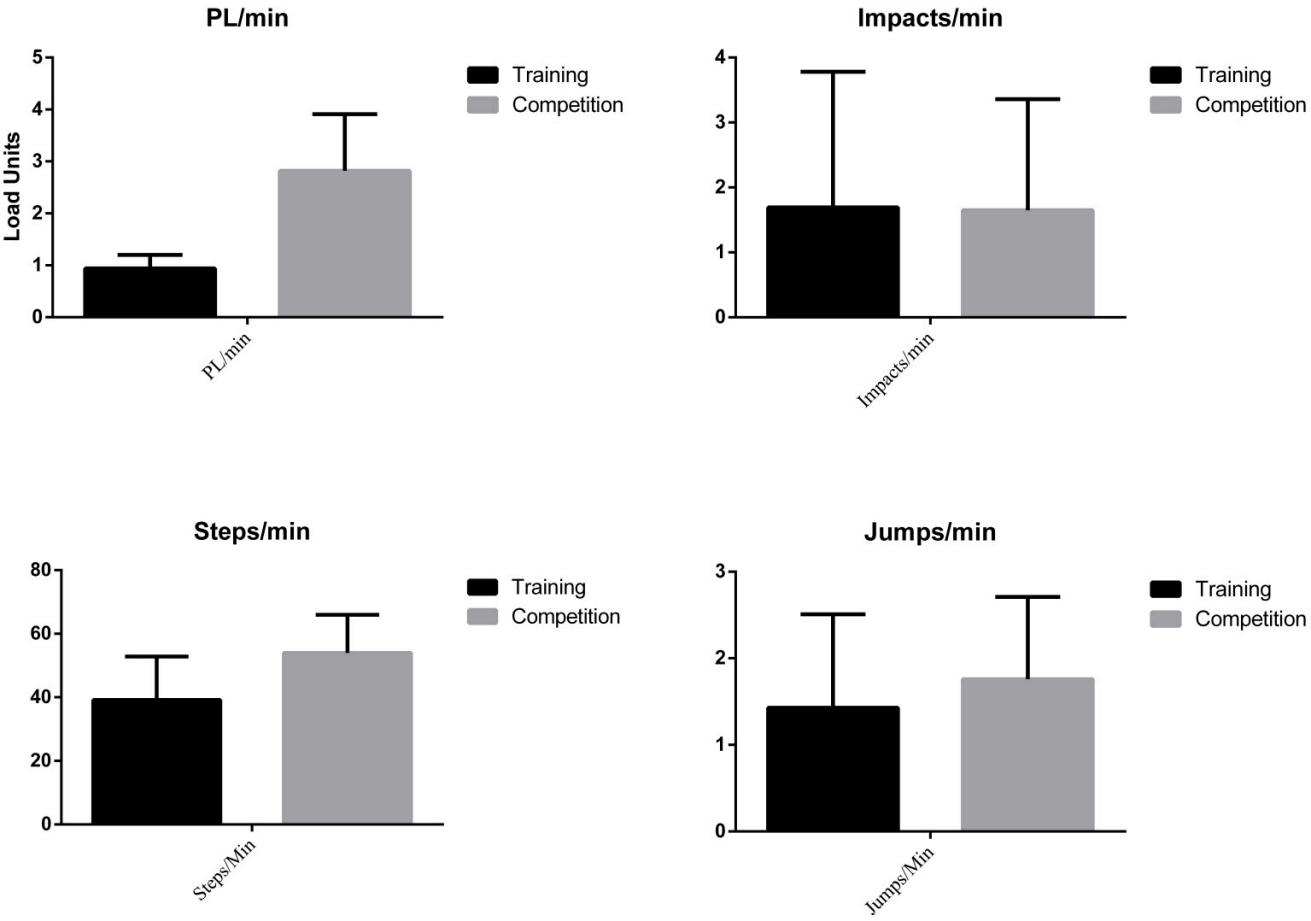
External load by Game Situation.

Besides, they reveal variations in the number of actions per minute when assessing the eTL variables (PL, Steps and Jumps per minute), except regarding the number of impacts per minute (Figure 4).

A large size effect was found in those variables that show a statistically significant difference. This effect size is >0.80 in the variables HRmáx, HRavg, %HRMáx, Z1, Z2, Z3, Z5, Z6, PL, and Steps per minute.

**Figure 5:**
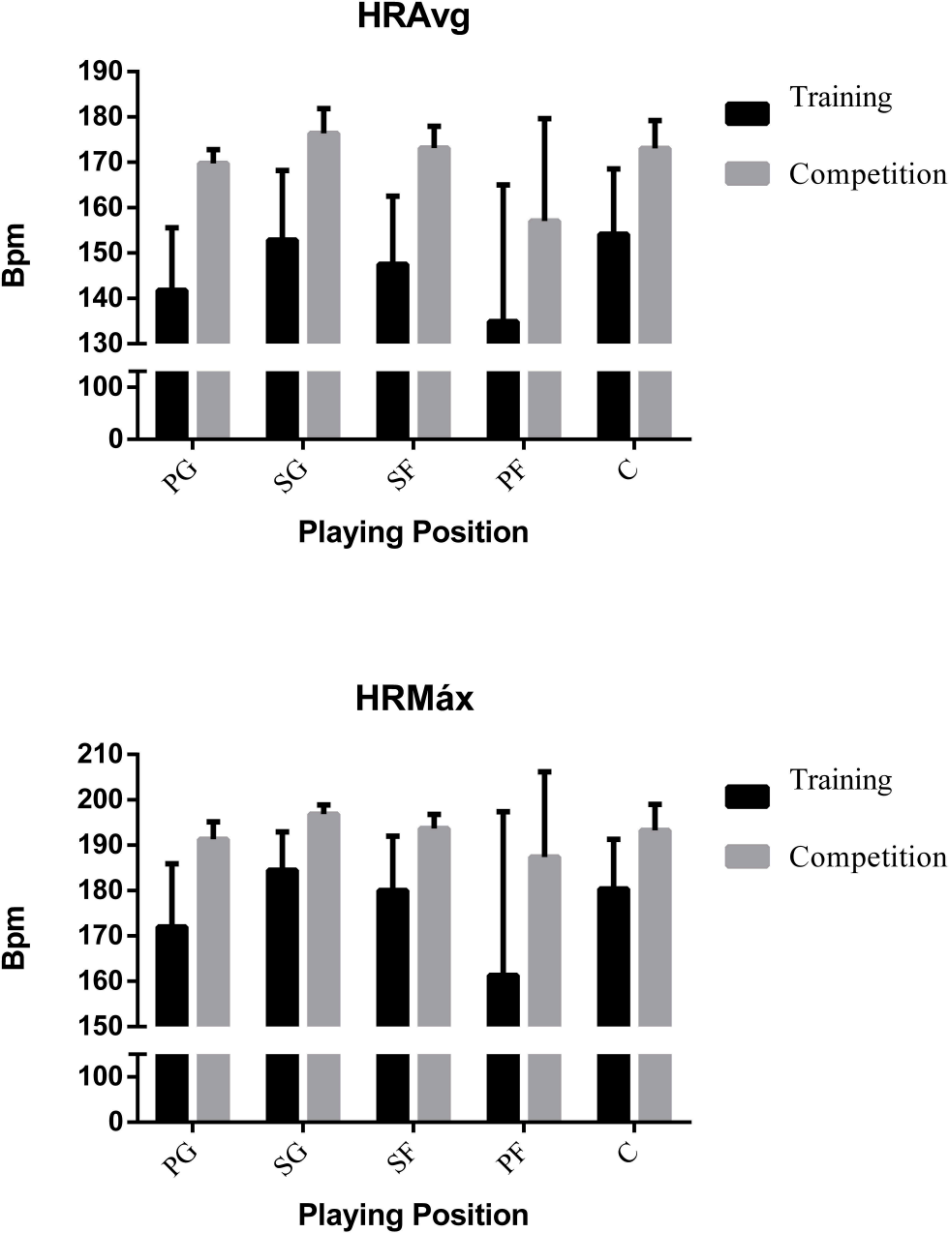
Internal load by playing position.

**Figure 6:**
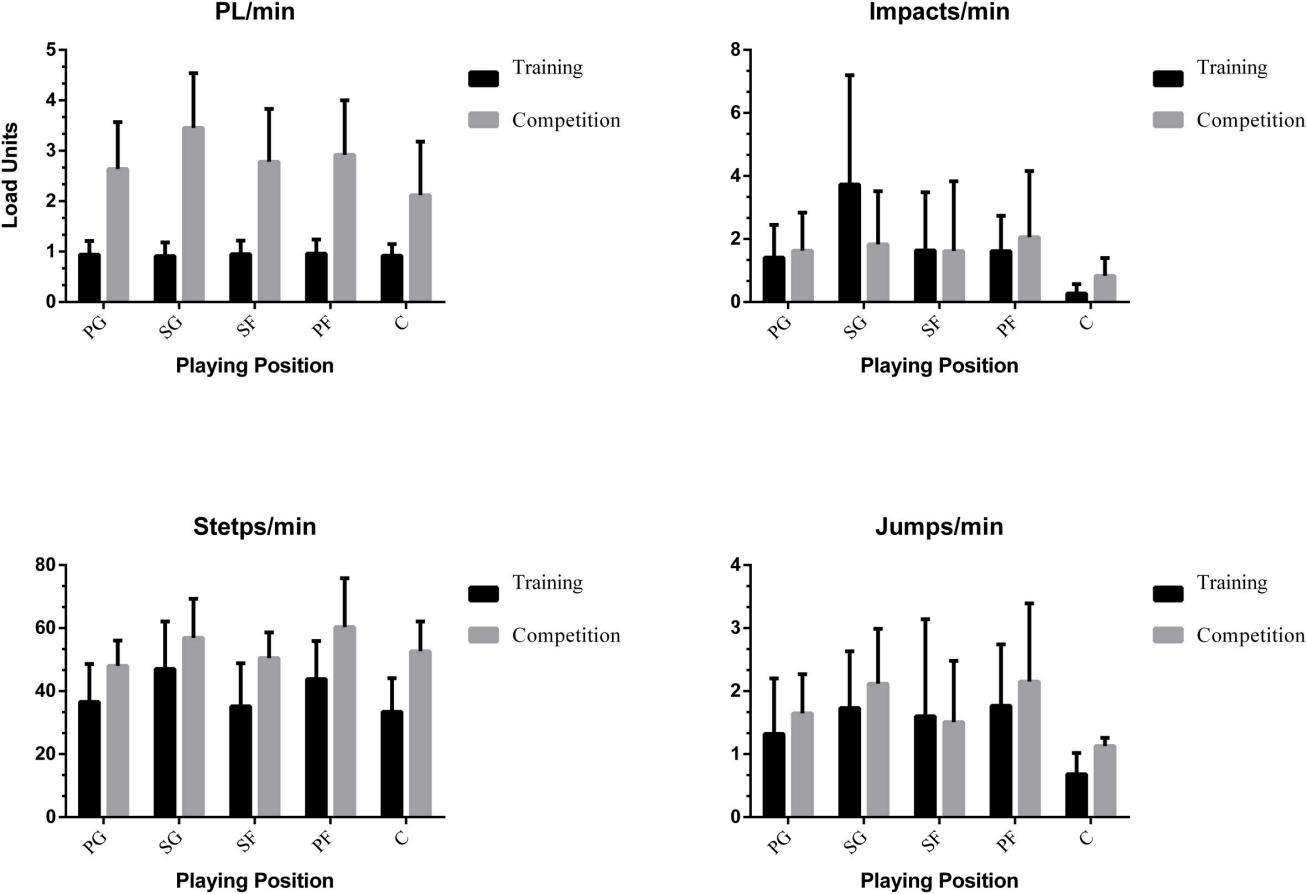
External load by playing position.

Secondly, Table 2 shows results from the analysis of differences between positions and the variables analyzed in training and competition. The results of the analysis on the basis of the specific playing position using a one-factor ANOVA and the effect size through Cohen's *d* show that there are statistically significant differences in the iTL, *Player Load* and *steps per minute* variables (*p* < 0.005), in all specific playing positions, comparing training and competition. In the case of the *impacts* and *jumps per minute* variables, there are statistically significant differences only for the Center role. The center is the player performing the least jumps and receiving the least impacts. The Shooting Guard supports a greater internal load, having values of 88.18% HRMax. In addition, the Power Foward is the one that accumulates the most external load, having values of 3.45 PL (Figures 5, 6). In addition, large effect sizes are shown except for the *impacts per minute* and *jumps per minute* variables, which are small.

**Table 2:** Inferential results as a function of the game situation and specific position.

		GS^*^	**Point guard** **(*n* = 16 competition)** **(*n* = 235 Training)**	**Shooting guard** **(*n* = 16 competition)** **(*n* = 235 Training)**	**Small forward** **(*n* = 16 competition)** **(*n* = 235 Training)**	**Power forward** **(*n* = 16 competition)** **(*n* = 235 Training)**	**Center** **(*n* = 16 competition)** **(*n* = 235 Training)**
iTL	HRMax	Training	171.97^**^	184.46^**^	180^**^	161.33^*^	180.34^**^
		Competition	191.33	196.83	193.67	187.43	193.25
	HRAvg	Training	141.82^**^	152.87^**^	147.52^**^	134.87^*^	154.18^**^
		Competition	169.76	176.36	173.14	157.04	173.09
	%HRMax	Training	70.91^**^	76.44^**^	73.76^**^	67.44^*^	77.09^**^
		Competition	84.88	88.18	86.57	78.52	86.55
eTL	PL/min	Training	0.94^**^	0.91^**^	0.95^**^	0.96^**^	0.92^**^
		Competition	2.64	3.45	2.78	2.92	2.12
	IMPACTS/min	Training	1.41	3.73	1.64	1.62	0.27^**^
		Competition	1.63	1.83	1.62	2.06	0.83
	STEPS/min	Training	36.58^**^	47.05^*^	35.14^**^	43.81^**^	33.44^**^
		Competition	48.03	56.91	50.51	60.28	52.65
	JUMPS/min	Training	1.32	1.73	1.6	1.77	0.68^**^
		Competition	1.65	2.12	1.51	2.15	1.13

* p < 0.05;

** p < 0.01;

*** *p < 0.000*.

## Discussion

The present investigation, as far as we know, is the first one to combine an internal and external load in women's basketball during training and competition according to a specific playing position. It was considered relevant to perform an inferential analysis according to playing position in order to guarantee specificity and individualized training principles, and differences were found. It has emerged that the load supported by the players during competition is higher, except for the number of impacts per minute. The players perform more steps and jumps per minute and their PL value is higher in competition. The shooting guard role is the one that has the highest %HR in competition, and its values of PL are greater than for the rest of the game positions. In the case of the power forward, she is the one who receives the most impacts per minute and performs more steps and jumps per minute. In addition, the competition imposes a greater physiological requirement than training.

When practicing basketball, the players have to experience similar demands to the ones experienced in competition, therefore, the trainers have to know and be able to reproduce them (Matthew and Delextrat, 2009; Conte et al., 2015; White and MacFarlane, 2015; Tee et al., 2016; Torres-Ronda et al., 2016). From the training standpoint it is useful to know if the workload has been below or above the real game reference loads, according to individual needs.

Regarding the load experienced by the athletes, there are different means that allow it to be defined in both training and sports competition. The monitoring of the external load measurements derived from triaxial accelerometers is currently considered a viable tool in team sports (Paul et al., 2010; Arruda et al., 2015). Besides, the evaluation of physical qualities through the use of different tests can also be useful to establish training programs, as well as to monitor it (Attene et al., 2016; Padulo et al., 2016). Previous studies have examined load using video Time Motion Analysis (Bishop and Wright, 2006; Scanlan et al., 2012; Hulka et al., 2014) and have presented controversial results in basketball studies as outcomes have varied according to the investigation (Bishop and Wright, 2006; Abdelkrim et al., 2007; Scanlan et al., 2012). Because of this, Arruda et al. (2015) suggest that using measurements derived from accelerometers could be an alternative, objective and reliable method to assess external load in training.

Player Load has been measured as a reliable and reproducible metric in the quantification of cumulative motion for indoor sports (Peterson and Quiggle, 2017). The use of inertial devices, such as accelerometers also makes it possible to analyze body impacts. This measurement represents an unspecific assessment of the amount and magnitude of specific high-intensity movements in basketball which cannot be registered by GPS (Weaving et al., 2014; Puente et al., 2017). The impacts per minute variable suggests the number of changes of direction, and actions involving the use of the body such as blocking, rebounding or defending (Barbero et al., 2014), which are an essential measure of physical load (Chambers et al., 2015). Per example, numerous short sprints might occur in successive different directions, therefore the development of a multi change of direction is expected to help fitness trainers and coaches for training evaluation purposes, among others (Padulo et al., 2016). The training with multiple changes of direction reproduces more closely the kinds of movements typical of basketball and should be preferred from an ecological point of view during training (Attene et al., 2016).

Physical fatigue using the variable PlayerLoad has been analyzed, but mental fatigue has not been taken into account. Marcora et al. (2009) establishes that subjects with mental fatigue qualify the perception of effort during exercise as significantly greater compared to the control condition. Therefore, it limits tolerance to exercise in humans through greater perception of effort. Besides (Padulo et al., 2018), confirm through their study the need for a good recovery by the players during breaks, waiting times, and substitutions since the increase in metabolic conditions, such as the increase in HR, decreases the jump performance.

Statistically significant differences between training and competition were found for the analyzed team regarding this kind of variables. Higher values were found in the PlayerLoad variable and the number of steps per minute in all the specific playing positions was higher in competition. In the case of impacts and jumps per minute, only the Center position revealed differences between training and sports competition. The Center players received more impacts and performed more jumps per minute in official matches. Therefore, the competition demands obtained a directly influence in the neuromuscular load of centers in relation to training sessions, not found this influence in the rest of playing roles. This effect could be produced due to a higher intensity of specific skills with physical contact (rebound, picks, to buttock down, etc.) during competition due to a higher level opposition. In this sense (Gabbett, 2016), claim that pre-competition sessions with a high load generate positive adaptations that reduce the risk of injury. So it is recommended to adjust the demands of competition to training.

Regarding the internal load, the specialized literature states that this kind of variables allow a more adequate control of the different responses of the organism toward training and competition (Makivić et al., 2013), therefore it is a vital complement when characterizing load. Compared to competition, Torres-Ronda et al. (2016) have shown that more similar cardiac responses to the ones in competition appear with 5 vs. 5 training, however, higher values were registered in men's basketball competition. Likewise, regarding women's basketball, Matthew and Delextrat (2009) obtained a HRAvg of 165 and a maximum of 170 in matches and Scanlan et al. (2012) obtained mean values of 162 bpm. In this study, HRAvg and HRMax during competition were 171 and 193 bpm while during training they were 145 and 175 bpm. The available literature shows lower values of HR, probably due to the sample studied. In Elite players have better fitness and conditioning, expressed as a lower HR in competition (Drinkwater et al., 2008), therefore it is necessary to specify the age, competitive level, gender and characters of the players.

Differences according to playing position are mainly due to the specificity and specialization of players, how they relate with their teammates or the needs of the competition (Sampaio et al., 2008, 2015). An understanding of these differences is essential for designing training sessions adequate for competitive demands. Some players may reach a higher volume of work for the entire session, while others may do less work overall but consistently reach higher intensities. Guards have to perform at high intensity on the whole court, whereas centers have to do so near the basket. These variations in demands are evident between playing positions and level of anaerobic and aerobic fitness (Abdelkrim et al., 2007; Tee et al., 2016). Monitoring volume and intensity during training and competition, and reporting data individually better than jointly appears to be essential (Howatson and Milak, 2009) for designing specific training sessions for the competitive demands of each player.

In this study, shooting guards are the players that face the greatest physical demand, both in 5 vs. 5 training and in competition. Their mean and maximum HR values are higher than the rest, as well as *Player Load*, impacts and steps per minute, while in jumps per minute maximum values are shown by the power forwards. According to Delextrat et al. (2015) shooting guards and small forwards cover more distance in offense without the ball than point guards, who demonstrate more ball control and have greater passing abilities. Regarding the internal load, differences were found in HRAvg and %HRMax between shooting guards and small forwards compared to power-forwards. Therefore, the outside players are the ones that show a higher physical demand through the HR. Based on this, Puente et al. (2017) affirm that the centers cover less distance and achieve a lower peak velocity than the outside players, revealing lower external and internal demands than the outside players.

Significant differences were found between the inside players (power forwards and centers). The centers have higher cardiac responses, while the power forwards cover greater distances, and perform more jumps and impacts per minute, both in training and competition. In other studies the difference between inside players and outside players has been noted (Torres-Ronda et al., 2016). Besides, players in these positions are clearly differentiated by their anthropometric characteristics in high-performance basketball (Ostojic et al., 2006), but not as clearly in trainees or non-professional players (Nikolaidis et al., 2014). It is possible that in this study, due to the non-professional status of the players, the anthropometric differences between the inside players imply differences in the physical demands, when previous research stated that they perform at a similar level (Delextrat et al., 2015; Torres-Ronda et al., 2016). Delextrat et al. (2015) characterize the inside players as performing more jumps and static efforts (blocking, positioning for the rebound…). In this study, significant differences were found between centers and other specific playing positions, showing less jumps per minute (1.13 jumps/min) and a lower *Player Load* variable (2.12 Player Load/min) supported by the inside players. However, power forwards achieved more jumps per minute and, with the shooting guards, were the ones that experienced a higher Player Load (2.29 Player Load/min). Inside players, due to their anthropometry, possibly do not need to perform some actions in play. For example, due to their physical dominance and height, centers do not need to jump for rebounding or blocking. On the other hand, power forwards record the highest number of impacts and jumps both in training and in competition. Therefore, this playing position would imply a greater effort during the game in order to achieve a better performance; hence, different conditioning training has to be planning according to playing position.

Competition presents higher demands regarding iTL and eTL compared to 5 vs. 5 training. Statistically significant differences were shown in every analyzed variable except for the number of impacts per minute. Differences were found in the demands generated by the different playing positions, but in all cases they were higher in competition. Training sessions usually do not exceed the time of an official match so the same requirements could apply, but, as observed, they are not of the same quality. Hence, the existing differences in the rest of the variables imply a higher game intensity and, thus, the needs of competition are not being met making it essential to generate an optimal load in the prior training so the athlete can face the demands of competition and be physically ready to compete without the risk of an injury.

## Conclusion

This is the first study to describe the internal and external load demands experienced by senior female basketball players during training and sports competition. Results show the importance of developing specific training plans and conditioning programs adapted to each player at every sport level. If training does not reach the specific demands of competition, a lower competitive performance will be achieved. In addition, rest and tapering periods should be established according to the load experienced by the athletes. Player Load is related with injury risk (Barreira et al., 2017). High intensity accelerations and decelerations are related with muscular damage and are not perceived by athletes until 24 h later (Howatson and Milak, 2009; Barreira et al., 2017). Regarding this point, the shooting guard and power forward experienced higher loads than the rest of the team; therefore, they would need more physical conditioning in training in order to maintain an optimal performance in competition. On the other hand, they also need resting periods to avoid the risk of injury.

Some of the main limitations of the work are those related to the size of the sample. Although a considerable number of games and training tasks have been used, only players from the same team participated, so it is unknown whether these physical profiles are generalizable to other teams of the same competitive level. Obtaining data in a massive way in a real situation of training and competition through the use of inertial devices is very expensive. On the one hand, the need to provide an IMU to each player. On the other hand, the limitations that some sport modalities establish, special permits being necessary. Therefore, a team that was willing to be monitored during a competitive period was contacted, while the official permission of the federation was obtained to be able to use it during the competition. The main strength has been to be able to use these devices in official competition matches, to verify the real load supported by the players in a specific context, in the field of play and not in the laboratory. Currently we are working on increasing the sample, of different categories and levels for a better definition of women's basketball in general.

This preliminary study was conducted with amateur players, and it is necessary to implement this study with professional players to check if this type of response is similar. It is necessary to carry out specific studies to individualize the results in each competitive level and age of training.

## Practical Applications

The results of this study provide the key to adapting the abilities that must be performed during training sessions according to player's positions. Thank these results. physical trainers and coaches will be able to adapt their training to what happens in competition, preparing players to face the same demands as those that are generated by real matches.

As for the internal load, the HRmáx obtained in training equals HRavg in matches. The players must work in higher areas of heart rate during training, mainly in Z4 and Z5. It could be achieved by interval exercises without full recovery, to work at the appropriate threshold. For example, a task of 5 vs. 5 in which each team makes at least three attacks and defenses, without stopping the game (dead ball, stops for fouls, out of bounds, field goal, etc.). As a rest interval, each team will make a free throw.

The eTL supported by the players in the competition is much higher than the one supported during the training. It is necessary for players to experience and train the load they have to endure in matches, mainly to prepare and prevent injuries. for this, the 5 vs. 5 tasks have to keep the players active and intense for a longer time and rest periods must be controlled so that they are not excessive. If necessary, rules can be established for different game positions to perform a minimum number of actions (such as jumps, changes of direction, contacts, etc.). For example:

- That it is not necessary to take off ball after goal to give continuity to the game and that the trainers establish the rest.
- That the ball touches the backboard or the rim to get point. Thus, we managed to increase the number of jumps and impacts to catch the ball by the inside players.
- That outside players have to make a certain number of changes of direction in each attack, with or without the ball.

## Author Contributions

MR: conceptualization, data collet, formal analysis, investigation, methodology, software, visualization, and writing original draft; JG-R: funding acquisition, supervision, writing original draft, writing review, and editing; SF: supervision, writing review, and editing. SI: supervision, writing review, and editing.

### Conflict of Interest Statement

The authors declare that the research was conducted in the absence of any commercial or financial relationships that could be construed as a potential conflict of interest.
